# Multifunctional and Transformable ‘Clickable’ Hydrogel Coatings on Titanium Surfaces: From Protein Immobilization to Cellular Attachment

**DOI:** 10.3390/polym12061211

**Published:** 2020-05-26

**Authors:** Tugce Nihal Gevrek, Aysun Degirmenci, Rana Sanyal, Amitav Sanyal

**Affiliations:** 1 Department of Chemistry, Bogazici University, Bebek, Istanbul 34342, Turkey; tcivan@gtu.edu.tr (T.N.G.); rana.sanyal@boun.edu.tr (R.S.); 2 Center for Life Sciences and Technologies, Bogazici University, Istanbul 34342, Turkey; aysun.degirmenci@boun.edu.tr

**Keywords:** click reactions, thiol-ene, thiol-maleimide, Diels–Alder, inverse-electron Diels–Alder, multi-functional, hydrogels, coatings, polymers, titanium interfaces

## Abstract

Multifunctionalizable hydrogel coatings on titanium interfaces are useful in a wide range of biomedical applications utilizing titanium-based materials. In this study, furan-protected maleimide groups containing multi-clickable biocompatible hydrogel layers are fabricated on a titanium surface. Upon thermal treatment, the masked maleimide groups within the hydrogel are converted to thiol-reactive maleimide groups. The thiol-reactive maleimide group allows facile functionalization of these hydrogels through the thiol-maleimide nucleophilic addition and Diels–Alder cycloaddition reactions, under mild conditions. Additionally, the strained alkene unit in the furan-protected maleimide moiety undergoes radical thiol-ene reaction, as well as the inverse-electron-demand Diels–Alder reaction with tetrazine containing molecules. Taking advantage of photo-initiated thiol-ene ‘click’ reactions, we demonstrate spatially controlled immobilization of the fluorescent dye thiol-containing boron dipyrromethene (BODIPY-SH). Lastly, we establish that the extent of functionalization on hydrogels can be controlled by attachment of biotin-benzyl-tetrazine, followed by immobilization of TRITC-labelled ExtrAvidin. Being versatile and practical, we believe that the described multifunctional and transformable ‘clickable’ hydrogels on titanium-based substrates described here can find applications in areas involving modification of the interface with bioactive entities.

## 1. Introduction

Titanium (Ti) and its alloys are widely used for orthopedic and dental implants, and amputation prosthesis due to their stability, excellent corrosion resistance, and good mechanical properties [1]. Although the oxide layer on titanium is considered to be biocompatible, it is known that bare titanium has poor integration with the surrounding bone environment since it adsorbs serum protein upon contact with blood or other body fluids [2,3], which often causes inflammation. Likewise, other implant materials, such as in vivo sensors and drug delivery devices, also require good biocompatibility with the surrounding biological environment. Therefore, modification of titanium-based materials for enhancing their long-term clinical performance has been an area of active research in biomaterial science.

Lately, modification of surfaces with polymeric materials has taken inspiration from biological organisms [4]. Especially, mimicking of mussel adhesive proteins by using dopamine-based building blocks has attracted significant interest. Mussels adhere to practically all types of inorganic and organic surfaces [5], including adhesion-resistant substrates such as poly(tetrafluoroethylene) (PTFE) [6]. The adhesive property of mussels stems from the amino acid composition of the proteins found near the plaque-substrate interface, which are rich in dihydroxyphenylalanine (DOPA) and lysines [7]. In addition to participating in reactions leading to bulk solidification of the adhesive [8,9], DOPA forms strong covalent and non-covalent interactions with various substrates [10]. It is well established that the catechol-based sub-unit of this adhesive protein binds strongly to a variety of metal/metal oxide surfaces [11,12,13,14]. 

Reactive polymeric coatings are at the heart of various biomedical applications, which range from sensing and delivery platforms to facilitate the adaptation of various implant materials. Widely used applications in biomedical sciences encompass coating of orthopedic materials, cardiovascular stents, antibacterial surfaces, drug delivery devices, tissue engineering scaffolds and biosensors [15,16]. Using hydrogels as a coating material is quite attractive since they provide a 3-dimensional (3D) soft material-based interface, whose properties can be tuned by the choice of the coating material and can be further augmented by appropriate functionalization [17,18,19,20,21,22,23]. For example, 3D-coatings can provide a higher capacity for protein immobilization [24], as well as offer a more homogenous and ‘natural’ environment to bio-macromolecules, compared to their flat 2D counterparts [25]. For example, Spring and coworkers fabricated *N*-hydroxysuccinimide containing 3D small-molecule microarrays on glass surface to obtain an amine reactive slide with high loading capacity, signal sensitivity and better spot morphology when compared with some commercially available slides and 2D slide containing the same reactive group and demonstrated that 3D hydrogel slides possessed higher loading capacity than various 2D slides [26]. 

Recent years have attracted a lot of interest in employing ‘click’ chemistry tools for multi-functionalization of polymeric materials, due to the efficient nature of these chemical transformations under relatively mild conditions [27,28]. In particular, the Huisgen 1,3-dipolar cycloaddition [29], strain-promoted (3+2) azide−alkyne cycloaddition [30,31], radical-mediated thiol-ene [32,33] and thiol-yne [34,35] reactions, Michael additions [36] and Diels–Alder [37,38,39,40] reactions have been widely used for immobilization of (bio)molecules on a wide variety of polymeric interfaces. Among all ‘clickable’ units, the maleimide functional group attracts attention due to their facile reaction with thiol- and furan-containing molecules under mild conditions [41,42,43,44]. We have earlier reported maleimide-containing polymers [45,46], surfaces [47,48,49,50] and bulk hydrogels [51] for biomolecular immobilization [52,53]. In a recent study, copolymers containing dopamine- and maleimide-containing monomers were used to obtain antibacterial coatings on titanium surfaces through attachment of anti-bacterial peptides [54]. Although the approach was successful to an extent, two critical challenges with this approach were realized. First, synthesis of such reactive copolymers is not trivial, and the presence of reactive groups makes such systems sensitive to long term storage. Secondly, the ultra-thin layer of coating realized through this approach limits the amount of loading of molecules of therapeutic interest in such systems. Thus, a methodology for coating titanium surfaces with thicker interfaces, and using monomers instead of polymers, will be of pragmatic significance. 

**Scheme 1:**
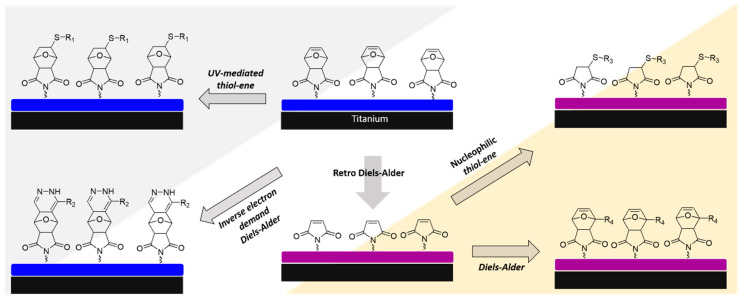
Schematic illustration of multi-functionalizable hydrogel layer on titanium substrates using various click reactions.

Herein, we report the preparation of a poly(ethylene glycol) (PEG) based furan-maleimide cycloadduct containing multifunctional hydrogel layer that is attached onto titanium surfaces in a robust manner using a dopamine methacrylamide based surface modifier. Furan-maleimide containing monomer was used as a versatile reactive monomer and poly(ethylene glycol) methyl ether methacrylate (PEGMEMA) was used to create the anti-biofouling matrix of the hydrogel. These monomers were photo-crosslinked on top of the methacrylamide bearing monolayer on titanium surfaces in the presence of a PEG-based cross-linker and a photo-initiator. Thus fabricated hydrogel coating could undergo radical thiol-ene and inverse-electron-demand Diels–Alder (iedDA) reactions with the strained oxanorbornene unit in the furan-maleimide cycloadduct. Furthermore, after removal of the furan units by simple heating under vacuum, the hydrogel surfaces gain ability to undergo functionalization through nucleophilic thiol-ene, as well as the thermo-reversible Diels–Alder cycloaddition reactions. Extent of hydrogel functionalization can be tailored by varying the amount of ‘clickable’ groups in the hydrogel. This readily obtained transformable ‘clickable’ hydrogel coating can be exploited for functionalization through four different metal catalyst-free ‘click’ transformations to obtain biofunctional interfaces (Scheme 1).

## 2. Materials and Methods 

### 2.1. Materials

Poly(ethylene glycol)methyl ether methacrylate (PEGMEMA, average *M*_n_ = 300 g/mol), poly(ethylene glycol) dimethacrylate (PEGDMA, *M*_n_ = 550 g/mol), 2,2-dimethoxy-2-phenylacetophenone (DMPA), biotin-benzyl-tetrazine and TRITC-conjugated ExtrAvidin were purchased from Sigma-Aldrich (Sigma, St. Louis, MO, USA). Dopamine methacrylamide (DMA) [55], furan-protected maleimide methacrylate (FuMaMA) monomer [56], BODIPY-SH [57], BODIPY-furan (BODIPY-F) [58] were synthesized according to literature procedures. The arginine-glycine-aspartate-cysteine (RGDC) peptide was obtained from Sigma (Sigma, St. Louis, MO, USA). Organic solvents were purchased from VWR (Darmstadt, Germany) and used as received without further purification. Silicon surfaces were coated with Ti through electron-beam evaporation. Prior to immobilization of DMA, titanium substrates were cleaned using a Novascan PSD Series UV/Digital Ozone System (Novascan Technologies, Inc., Boone, IA, USA) for 30 min. Thickness of the hydrogel coating was measured using a scanning electron microscopy instrument, ESEM-FEG/EDAX Philips XL-30 (Philips, Eindhoven, The Netherlands). Blak-Ray^®^ B-100 AP/R High Intensity UV lamp (100 Watt/365 nm) (Fisher Scientific Ltd., Hampton, NH, USA) was used for photo-polymerization. Attenuated total reflectance Fourier transform infrared (ATR-FTIR) spectroscopy was performed on a Thermo Scientific Nicolet 380 FTIR spectrophotometer (Thermo Fisher Scientific, Waltham, MA, USA). Fluorescence microscopy images of samples on titanium surfaces were recorded on a Zeiss Observer. Z1 fluorescence microscope (Carl Zeiss, Jena, Germany). BODIPY derivatives and Alexa Fluor 488 were visualized by filter set 38, TRIT-C conjugated ExtrAvidin was visualized by filter set 43 and DAPI was visualized by filter set 49.

### 2.2. Representative Fabrication of Hydrogel Coating

Prior to hydrogel coating, ozone plasma-cleaned titanium substrates were immersed in the solution of DMA in methanol (1 mM, 100 mL) for 18 h at room temperature to obtain a reactive adlayer coating. They were then washed with methanol and dried under a stream of nitrogen. For the synthesis of 10% FuMaMA containing hydrogel H1, FuMaMA (20 mg, 0.069 mmol), PEGDMA (37.78 mg, 0.069 mmol), PEGMEMA (164.7 mg, 0.550 mmol) and DMPA (8.8 mg, 0.034 mmol) were dissolved in DMF (250 µL). Ten microliters of hydrogel precursor solution were spread onto 1 × 1 cm^2^ titanium surface via spin-coating at 500 rpm for 18 s. The solution was then covered with a microscope cover glass and placed under UV light at a distance of 10 cm for 30 min. Lastly, the cover glass was taken off and the hydrogel layer on surfaces was washed with copious amount of DMF and THF, and dried under a gentle stream of nitrogen. Hydrogels H2 and H3 were synthesized using the abovementioned protocol by increasing the FuMaMA monomer ratio to 30% and 50% and keeping the molar amount of cross-linker, DMPA and molarity of the solution as constant. 

### 2.3. Activation of Maleimide Functional Groups

Furan-protected maleimide-containing hydrogel layers on titanium surfaces were placed in a preheated vacuum oven at 120 °C for 30 min. Heating was then switched off and surfaces were kept under vacuum for 120 min to cool down to room temperature before exposing to ambient atmosphere.

### 2.4. Functionalization with BODIPY-F and Re-Functionalization Using the Diels–Alder/Retro Diels–Alder Reaction Sequence

Hydrogel coated surfaces were incubated in a solution of BODIPY-F (0.1 mg/mL in toluene). Hydrogels were incubated in the solution for 18 h in dark. Subsequently, non-reacted dyes were rinsed off using toluene and THF. In order to remove BODIPY-F from hydrogels, surfaces were heated up to 110 °C for 18 h in DMF and washed with a copious amount of DMF and THF. 

### 2.5. Functionalization with BODIPY-SH via Nucleophilic Thiol-Ene Reaction

Hydrogel coated surfaces were placed in a vial containing BODIPY-SH solution (1 mg/mL in DMF) for 18 h in dark at room temperature. After conjugation, the dye solution was removed, and the hydrogel sample was gently rinsed with copious amounts of DMF.

### 2.6. Functionalization with RDGC via Nucleophilic Thiol-Ene Reaction

Hydrogel coated titanium surfaces were placed into a solution of RGDC (1 mg/mL in DMF. After incubation for 18 h, the peptide solution was removed, and substrates were washed with DMF, and dried under a gentle flow of N_2_ gas.

### 2.7. Functionalization of Hydrogels with BODIPY-SH via Radical Thiol-Ene Reaction

BODIPY-SH (1 mg, 2.38 × 10^−3^ mmol) and DMPA (0.12 mg, 0.476 × 10^−3^) were dissolved in THF. The hydrogel layer on the titanium was soaked with 10 µL of the dye solution and the surface was exposed to UV light for 5 min through a photo-mask placed on the hydrogel. Subsequently, the hydrogel layer was washed with a copious amount of THF to remove all unbound materials.

### 2.8. Functionalization of Hydrogels with Biotin-benzyl-tetrazine via Inverse-Electron-Demand Diels–Alder Reaction

The hydrogel coated substrate was incubated in a solution of Biotin-benzyl-tetrazine in 1:1 THF/ MeOH mixture (0.5 mg/mL). After 18 h, the samples were washed with THF and MeOH and dried under a gentle stream of N_2_. A solution of TRITC-ExtrAvidin (20 μL, 0.1 mg/mL in PBS) was dropped on the biotinylated hydrogel samples. The samples were placed for 30 min in a dark place, and then they were gently rinsed with copious amounts of PBS and water.

### 2.9. Cell Culture on Peptide Functionalized Hydrogels

NIH/3T3 mouse fibroblast cells which were grown in 5% CO_2_ containing atmosphere at 37 °C were cultured in Dulbecco’s Modified Eagle’s Medium (DMEM) media supplemented with 10% fetal bovine serum (FBS) (PAN Biotech). NIH/3T3 mouse fibroblasts were seeded onto prepared hydrogel coated surfaces with a density of 8 × 10^3^ cells/cm^2^. After dropping the cell suspensions onto coated slides, they were incubated at 37 °C in 5% CO_2_ containing atmosphere for 3 h. Afterward, cell media (0.5 mL) was added into each well containing hydrogel coated surfaces. After incubation (24 h), cell media was removed, and surfaces were washed with 1× PBS (3 times) and cells were fixed with 3.7 % formaldehyde solution for 10 min at room temperature. For staining filamentous actins (F-actins), first, cells were incubated in 0.1% Triton X-100 in PBS for 5 min, then after washing with 1× PBS (3 times), they were incubated in Alexa Fluor 488 phalloidin solution (5 units/mL concentration containing 1% bovine serum albumin (BSA) in 1× PBS) for 20 min at room temperature. Subsequently, cell nuclei were stained with DAPI for 10 min at room temperature. Resulting images of cells attached onto hydrogels were taken using a fluorescence microscope and processed via Zeiss AxioVision software (Carl Zeiss, Jena, Germany).

## 3. Results and Discussion

**Figure 1:**
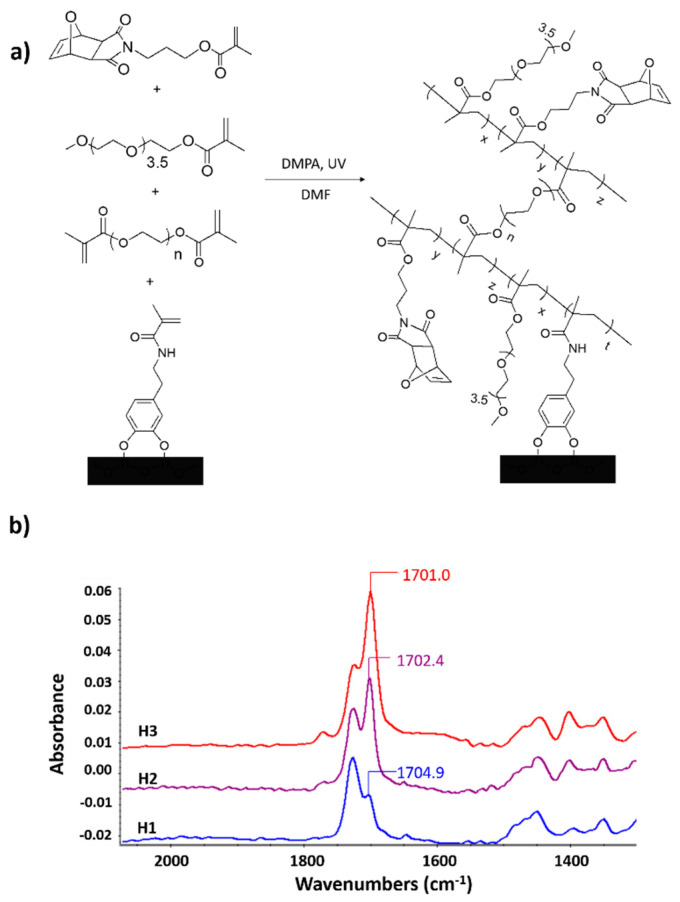
(**a**) Synthesis of FuMaMA containing latent reactive hydrogels on titanium. (**b**) ATR-FTIR spectra of the hydrogels.

Reactive hydrogel coatings on titanium surfaces were fabricated using a furan-protected maleimide methacrylate (FuMaMA) monomer and poly(ethylene glycol)methyl ether methacrylate (PEGMEMA) via photo-polymerization in the presence of poly(ethylene glycol) dimethacrylate (PEGDMA) as a crosslinker and 2,2-dimethoxy-2-phenylacetophenone (DMPA) as a photo-initiator (Figure 1a). To ensure robust attachment of the hydrogel coatings onto the underlying titanium surfaces, dopamine methacrylamide (DMA) was utilized as an adlayer. The catechol group on this molecule provides the necessary anchoring onto the titanium surfaces, while the methacrylate group allows covalent bonding to the polymeric structure of the hydrogel coating. Importantly, the fabrication protocol is quite pragmatic. The hydrogel precursor containing monomers, crosslinker and photo-initiator was spin-coated on the modified titanium surface, covered with a microscope cover glass and irradiated with ultraviolet light for 30 min. By altering the FuMaMA monomer amount as 10%, 30% and 50%, hydrogels H1, H2 and H3 with different functional group densities were synthesized on modified titanium surfaces, respectively. Thickness of hydrogel coatings were around 20 µm, as determined using ESEM (Figure S1).

**Figure 2:**
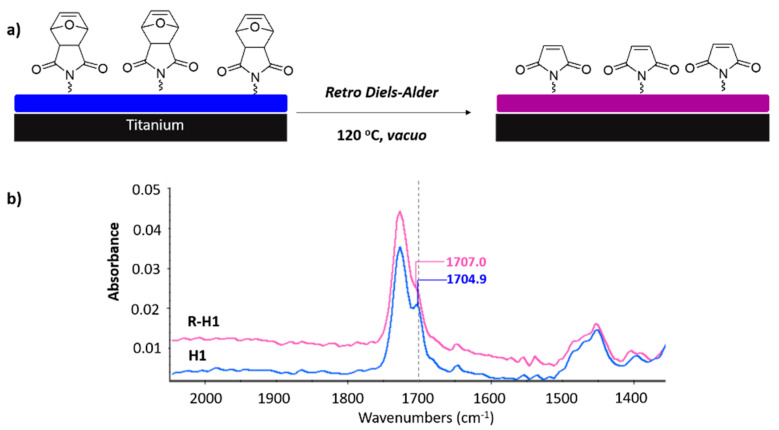
(**a**) Removal of furan groups via retro Diels–Alder reaction. (**b**) ATR-FTIR spectra of hydrogel H1 before and after thermal treatment via retro Diels–Alder reaction.

Incorporation and control over density of FuMaMA units with changing feed ratio of monomers in the precursor solution was confirmed using ATR-FTIR analysis. The FTIR spectra showed a C=O stretching band belonging to ester groups at ~1727 cm^−1^ for all surfaces. In addition, the spectra revealed the presence of the out-of-phase C=O stretching vibration at 1704.9, 1702.4 and 1701.0 cm^−1^ corresponding to furan-maleimide cycloadduct units of hydrogel H1, H2 and H3, respectively. In addition, weak bands at 1765.6, 1770.8, 1772.5 cm^−1^ for H1, H2 and H3, respectively, were assigned to the in-phase C=O stretching vibration of maleimide units (Figure 1b). As expected, the FTIR spectra showed an increase in the intensity of those stretching vibrations with an increase in the amount of FuMaMA monomer in gelation feed. Thermal treatment unmasks the maleimide groups to their thiol- and diene- reactive form and yields hydrogels ready for functionalization with nucleophilic thiol-ene and Diels–Alder reactions (Figure 2a). Successful the retro Diels–Alder reaction to unmask maleimide groups was confirmed using ATR-FTIR, from the shift of C=O stretching vibration band to a higher wavenumber (Figure 2b), similar to previous reports in the literature [47,49,59].

**Figure 3:**
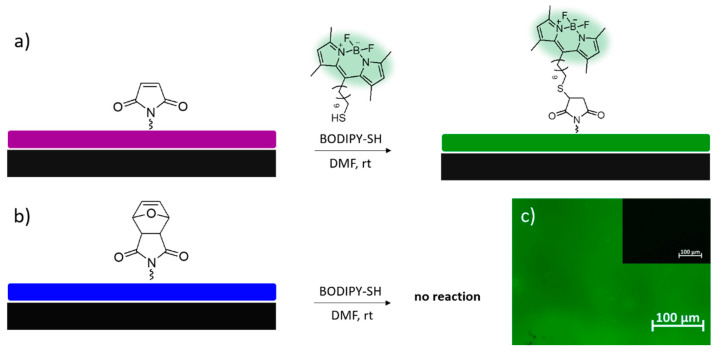
(**a**) Scheme of immobilization of BODIPY-SH onto hydrogel R-H1, (**b**) control experiment with protected maleimide containing surface, and (**c**) and fluorescence microscope images of dye immobilization (inset: control reaction).

After deprotection of maleimide units via the retro Diels–Alder reaction, a fluorescent dye, namely, BODIPY-SH, was immobilized onto the surface in order to demonstrate facile functionalization of these interfaces using a nucleophilic thiol-ene reaction (Figure 3). Thiol containing dye solution was prepared in DMF and activated and non-activated surfaces were incubated in this solution for 18 h. Thereafter, surfaces were washed with DMF and THF to remove unbound dye, and visualized under a fluorescence microscope. Appearance of bright green fluorescence indicated successful conjugation of the fluorescent dye (Figure 3c). As expected, due to the lack of nucleophilic attack of thiol-containing dye to the furan-protected maleimide unit, no fluorescence was observed for hydrogels prior to deprotection (Figure 3c inset).

**Figure 4:**
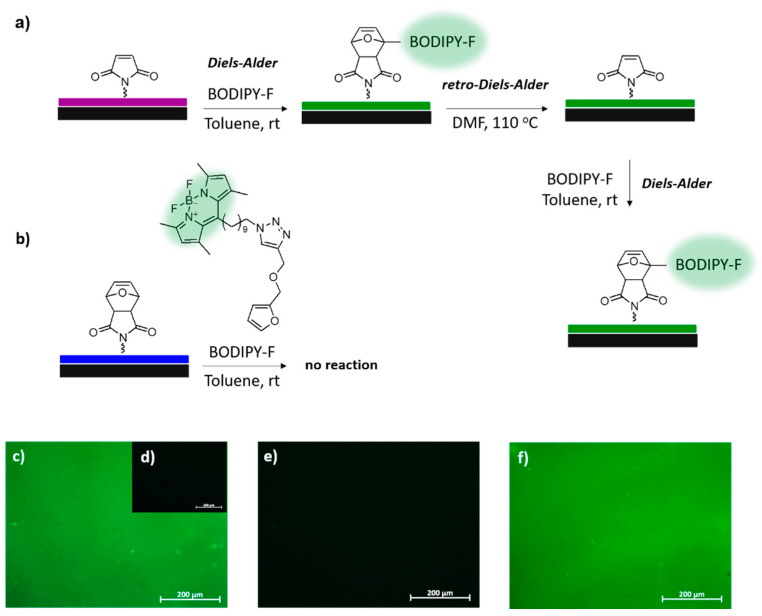
(**a**) BODIPY-F reversible conjugation sequence, (**b**) control reaction. Fluorescence microscopy images of (**c**) dye conjugated hydrogel; (**d**) control experiment; (**e**) hydrogel after dye de-conjugation and (**f**) hydrogel after dye re-conjugation.

Functionalization of hydrogels through the Diels–Alder reaction with diene bearing molecules was evaluated using a furan-containing BODIPY dye (BODIPY-F) (Figure 4). Hydrogel H1 obtained after the retro Diels–Alder reaction, namely R-H1, was immersed in toluene containing BODIPY-F at room temperature for 18 h. After washing with copious amounts of THF to remove any unbound dye, bright green fluorescence was observed when thus modified surface was analyzed using fluorescence microscopy. When hydrogel H1, where the maleimide units had not been unmasked was used instead of hydrogel R-H1, no significant fluorescence was detected, which suggests that functionalization was realized through the Diels–Alder reaction (Figure 4d). It is well known that the Diels–Alder linkage between maleimide and furan is thermo-reversible, hence, as expected, the dye is removed upon heating the BODIPY-F conjugated hydrogel surfaces to 110 °C in DMF. Lack of any appreciable fluorescence in the hydrogel indicated successful release of the dye (Figure 4e). In order to demonstrate that absence of fluorescence signal stems from removal of the furan-containing dye and not dye decomposition, we incubated the obtained hydrogel layer in dye solution and showed re-functionalization of the same surface (Figure 4f). This supports the fact that indeed the removal of dye proceeds through the retro Diels–Alder reaction which simultaneously creates the active maleimide units in the hydrogel, which can be used for subsequent re-functionalization.

**Figure 5:**
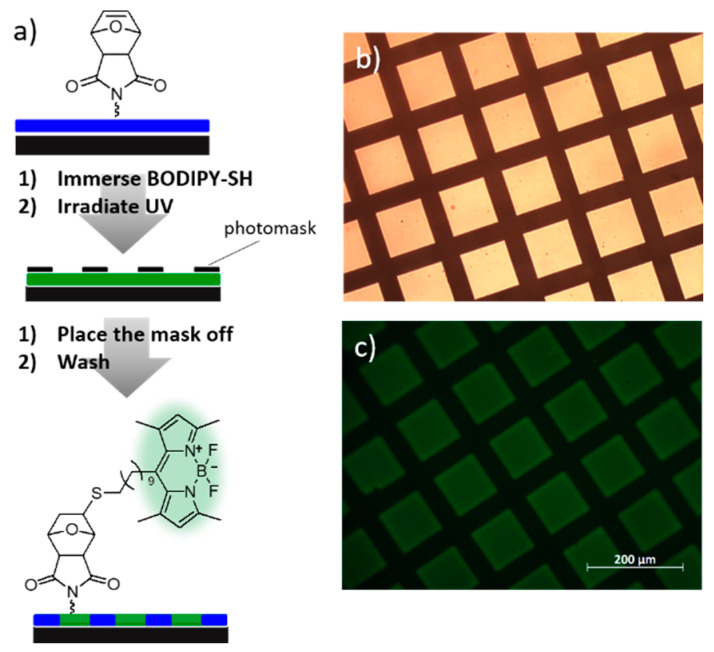
(**a**) Schematic illustration of patterned immobilization via thiol-ene reaction. (**b**) Optical microscopy image of the photo-mask used, and (**c**) fluorescence microscope image of BODIPY patterns on hydrogel H1.

The presence of bicyclic strained alkenes such as those present in the furan-maleimide cycloadduct can allow functionalization of thus prepared hydrogel coating by two different ‘click’ reactions, namely, the radical thiol-ene and inverse-electron-demand Diels–Alder (ieDDA) reactions. Ability to functionalize at the cycloadduct stage circumvents the need for further activation to the maleimide group. Thus we investigated the conjugation of thiol and tetrazine containing molecules as prepared hydrogel surfaces, without thermal activation. While the maleimide group has the advantage of rapid functionalization through the thiol-maleimide chemistry, functionalization in a spatially controlled manner is difficult. The photo-chemically initiated radical thiol-ene reaction allows functionalization in a spatially controlled manner. To demonstrate that this is a method of spatially controlled functionalization is applicable to as-prepared hydrogel coating and patterned immobilization of fluorescence dye molecule BODIPY-SH was performed (Figure 5). The hydrogel layer on titanium was soaked with BODIPY-SH and DMPA solution in THF and a photo-mask was placed onto the hydrogel (Figure 5a). After exposing the surface to the UV light for 5 min, photo-mask was removed, and excess dye was washed off using copious amounts of THF. Fluorescence microscopy images revealed that dye molecules were attached to oxanorbornene units on the exposed part of the surface while the unexposed parts remained unmodified (Figure 5b).

**Figure 6:**
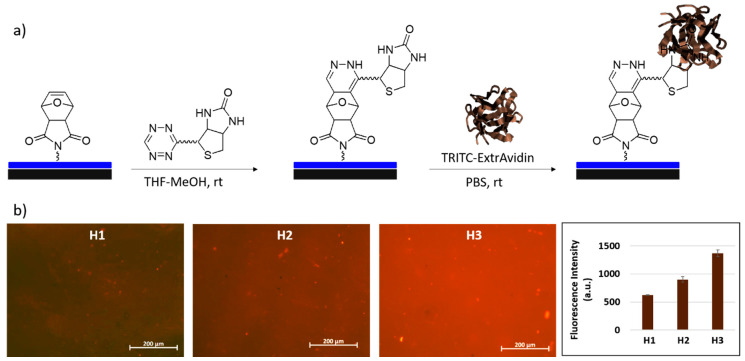
(**a**) Scheme of conjugation of oxanorbornene containing hydrogels with biotin-benzyl-tetrazine and following Tritc-ExtrAvidin immobilization; (**b**) fluorescence microscope images of Tritc-ExtrAvidin immobilized hydrogels and relative fluorescence intensity graph.

An alternative functionalization was also explored, since it is known that the oxanorbornene unit of the furan-maleimide cycloadduct reacts irreversibly with the tetrazine to yield a dihydropyrazine product. The furan-protected maleimide unit contains a strained electron rich dienophile—a highly reactive functional group that undergoes iEDDA reaction with molecules containing the tetrazine moiety. A tetrazine-containing biotin was utilized to functionalize the prepared hydrogel coatings (Figure 6a). Thereafter, incubation of Tritc-ExtrAvidin on biotinylated surfaces resulted in efficient immobilization of the protein, as deduced from their red fluorescence, upon analysis using a fluorescence microscope. One can anticipate that an increase in the amount of the strained alkene containing oxanorbornene unit within the hydrogel should allow increased extent of biomolecular immobilization. Indeed, it was observed that fluorescence intensities of the protein immobilized hydrogels increased with an increasing amount of oxanorbornene in the hydrogels (Figure 6b). This experiment clearly demonstrates that the extent of functionalization can be controlled by tuning the amount of the reactive monomer in the hydrogel coating. As a control experiment, non-biotinylated hydrogel H2 was incubated in a solution containing Tritc-ExtrAvidin. Fluorescence microscopy analysis revealed absence of any significant fluorescence, thus indicating that the hydrogel matrix is anti-biofouling and thus does not exhibit appreciable non-specific interactions with the protein (Figure S2). This type of efficient binding with specificity and minimal non-specific adsorption is an inherent requirement for utilization of such interfaces as biosensing platforms.

**Figure 7:**
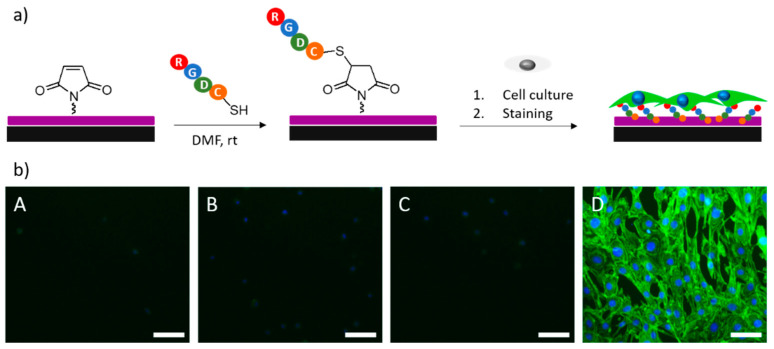
(**a**) Scheme of conjugation of cell-adhesive peptide RGDC and subsequent cell adhesion. (**b**) Fluorescence microscopy images of scaffolds showing Alexa Fluor 488 phalloidin (green) and DAPI (blue) staining of NIH/3T3 mouse fibroblasts after 24 h on A) hydrogel without FuMaMA; B) FuMaMA containing hydrogel; C) maleimide-containing hydrogel and D) RGDC-functionalized hydrogel (scale bar: 50 μm).

Hydrogel coatings on implant surfaces play an important role in promoting integration with cells and tissues, as well as drug depots to inhibit post-implantation implant rejection. While PEG-based materials have several attractive features that have made them a biomaterial of choice, their anti-biofouling nature is also well documented [60,61]. While the later attribute can be very beneficial in sensing platforms as demonstrated through previous examples, this feature can hamper their integration with cellular interfaces. It is well known that cellular adhesion on surfaces can be controlled by modifying them with specific peptides that have affinity for the receptors on cell surfaces. Among several peptides, the RGD sequences are well known to recognize the integrin receptors and promote attachment of cells. Hence, to demonstrate that while the ‘clickable’ hydrogel coating described here does not have any inherent affinity for attachment of cells but can be easily tailored to promote cellular adhesion, we attached thiol-containing RGDC peptides on R-H1 surfaces via Michael addition (Figure 7). Mouse fibroblast L929 cells which are known to have affinity for this peptide, were seeded onto these modified surfaces, as well as on unmodified maleimide containing hydrogel coatings (R-H1). Hydrogels without the FuMaMA monomer, as well as the non-activated FuMAMA containing surfaces were also used as controls. After incubation for 24 h, the actins and nuclei of the cells were stained. A significant difference was observed between the RGDC immobilized and control hydrogels. Due to the antifouling character of PEG, only few cells adhered on the hydrogel which had not been modified via RGDC, while clearly an abundance of cells adhered onto the RGDC peptide conjugated surface. From the green fluorescence of the stained actin filament, it was clear that the cells on peptide conjugated surfaces were spreading on the substrate.

## 4. Conclusions

In conclusion, furan-protected maleimide-containing multi-clickable biocompatible hydrogel layers on titanium surfaces were fabricated. To preserve the maleimide functional group during the polymerization reaction, it needed to be protected with furan as a thermo-reversible cycloadduct. After removal of the furan units, facile functionalization of hydrogels was demonstrated using Diels–Alder and nucleophilic thiol-ene reactions. Furthermore, those hydrogels could be functionalized using the furan-maleimide cycloadduct due to their reactivity towards radical thiol-ene and inverse-electron-demand Diels–Alder reactions. Taking advantage of site-specific UV thiol-ene click reaction, we showed patterned immobilization of the fluorescent dye BODIPY-SH through a photo-mask. Lastly, we demonstrated that the extent of functionalization on hydrogels can be controlled by attachment of biotin-benzyl-tetrazine followed by immobilization of TRITC-labelled ExtrAvidin. Being versatile and practical, we believe that described multi-functional hydrogels on titanium-based substrates described here can find applications in areas involving modification of the interface with bioactive entities.

## Supplementary Materials

The following are available online at https://www.mdpi.com/2073-4360/12/6/1211/s1, Figure S1: Thickness of hydrogel coating H1 was determined using ESEM, Figure S2: a) Control experiment with non-biotinylated H2 and related fluorescence microscope image.
