# Supplementary material for: Multifunctional and Transformable ‘Clickable’ Hydrogel Coatings on Titanium Surfaces: From Protein Immobilization to Cellular Attachment
Source: Polymers (Basel). 2020 May 26;12(6):1211. doi: 10.3390/polym12061211 (PMC7362003; doi:10.3390/polym12061211)
Supplement: Supplementary file 1 [file polymers-12-01211-s001.pdf]

Multifunctional and Transformable 'Clickable' Hydrogel Coatings on Titanium Surfaces: From Protein Immobilization to Cellular Attachment

Tugce Nihal Gevrek [1], Aysun Degirmenci [2], Rana Sanyal [1,2] and Amitav Sanyal [1,2,*]

[1] Department of Chemistry, Bogazici University, Bebek, Istanbul, 34342, Turkey; e-mail@e-mail.com

[2] Center for Life Sciences and Technologies, Bogazici University, Istanbul, 34342, Turkey; e-mail@e-mail.com

* Correspondence: amitav.sanyal@boun.edu.tr; Tel.: +90-212-359-7613; Fax: 90-212-287-2467

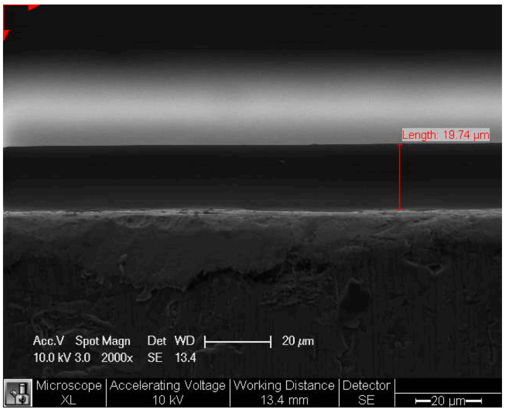

Figure S1. Thickness of hydrogel coating H1 determined using ESEM.

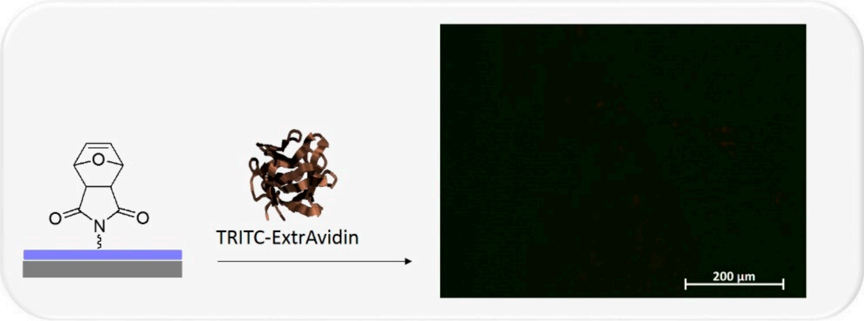

Figure S2. Control experiment with non-biotinylated H2 and related fluorescence microscopy image.
